# Investigation of mitochondrial-derived plastome sequences in the *Paspalum* lineage (Panicoideae; Poaceae)

**DOI:** 10.1186/s12870-018-1379-1

**Published:** 2018-08-03

**Authors:** Sean V. Burke, Mark C. Ungerer, Melvin R. Duvall

**Affiliations:** 1 Department of Biological Sciences and Plant Molecular and Bioinformatics Center, Northern Illinois University, 1425 W. Lincoln Hwy, DeKalb, IL 60115-2861 USA; 2 Division of Biology, Kansas State University, 1717 Claflin Rd, Manhattan, KS 66506-4900 USA

**Keywords:** Grasses, Inserts, mtDNA, Next generation sequencing, *Paspalum*, Poaceae, ptDNA

## Abstract

**Background:**

The grass family (Poaceae), ca. 12,075 species, is a focal point of many recent studies that aim to use complete plastomes to reveal and strengthen relationships within the family. The use of Next Generation Sequencing technology has revealed intricate details in many Poaceae plastomes; specifically the *trnI* - *trnL* intergenic spacer region. This study investigates this region and the putative mitochondrial inserts within it in complete plastomes of *Paspalum* and other Poaceae.

**Results:**

Nine newly sequenced plastomes, seven of which contain an insert within the *trnI* - *trnL* intergenic spacer, were combined into plastome phylogenomic and divergence date analyses with 52 other species. A robust *Paspalum* topology was recovered, originating at 10.6 Ma, with the insert arising at 8.7 Ma. The alignment of the insert across *Paspalum* reveals 21 subregions with pairwise homology in 19. In an analysis of emergent self-organizing maps of tetranucleotide frequencies, the *Paspalum* insert grouped with mitochondrial DNA.

**Conclusions:**

A hypothetical ancestral insert, 17,685 bp in size, was found in the *trnI* - *trnL* intergenic spacer for the *Paspalum* lineage. A different insert, 2808 bp, was found in the same region for *Paraneurachne muelleri*. Seven different intrastrand deletion events were found within the *Paspalum* lineage, suggesting selective pressures to remove large portions of noncoding DNA. Finally, a tetranucleotide frequency analysis was used to determine that the origin of the insert in the *Paspalum* lineage is mitochondrial DNA.

**Electronic supplementary material:**

The online version of this article (10.1186/s12870-018-1379-1) contains supplementary material, which is available to authorized users.

## Background

The grass family (Poaceae) comprises 12 subfamilies containing ca. 12,075 species [1]. Recent studies have delved into the phylogenomic framework of Poaceae using complete plastid genomes (plastomes) as a genomic marker; many of which have obtained plastomes by means of Next Generation Sequencing (NGS) methods. NGS technology has allowed for more extensive and deeper sampling within the grass subfamilies [2–15], tribes [16–19] and even genera (*Alloteropsis*: [20, 21]; *Zea*: [22]). This in-depth sampling strategy has allowed researchers to discover unique features of poaceous plastomes.

One such feature is the transfer of mitochondrial DNA (mtDNA) to plastome DNA (ptDNA). This type of mutational event was originally thought to be nonexistent or extremely rare [23–25]. The first reported case of mtDNA transfer to ptDNA was discovered in *Daucus carota* [26] and then a second discovery in *Asclepias syriaca* [27]. With both discoveries occurring within eudicots, it was not until Wysocki et al. [14], that mtDNA to ptDNA transfers were found in monocots.

The first discovery in monocots implicated transfers in two bamboos, *Eremitis* sp. and *Pariana radiciflora*, from the same subtribe, Parianinae. The inserts within *Eremitis* sp. and *Pariana radiciflora* were roughly 5 kilobase pairs (kbp) and 2.7 kbp, respectively, and with highly similar sequence for a 2.7 kbp section [14]. This was later expanded on with the inclusion of two more Parianinae, *Pariana* sp. and *P. campestris* [9], which also contained homologous inserts in the same region, the *trnI* - *trnL* intergenic spacer (IGS). While there have been other discoveries of mtDNA in ptDNA inserts found in grasses [*Triticum monococcum*: 11] and other eudicots [*Lavandula angustifolia*, *Orobanche californica*, *Scutellaria baicalensis*: 28], the placement of the insert within the *trnI* - *trnL* IGS has been of special interest.

The authors of a recent study [3] investigated this region, and found putative mtDNA inserts within the *trnI* - *trnL* IGS in two species of *Paspalum*. These inserts contained no similarity to each other, nor any similarity to the species of Parianinae in the same region. Their conclusions were either that multiple inserts within *Paspalum* arose independently of each other or that the two events were portions of a larger insert. If a mechanism for such intercompartmental DNA transfers were known, the taxonomic distribution of such events might be more evident. However, no such mechanism has been found [25]. This study will build on the previous study of Burke et al. [3], by increased sampling of species in *Paspalum*, to determine the origin, mechanism, and timing of the insertions found in plastomes of *Paspalum*.

## Methods

### Sampling

Species of *Paspalum* from another study [29] that were found to be closely related to those with and without the mtDNA insert [3] were selected for further analysis in this paper. Based on these criteria, eight new *Paspalum* plastomes (Table 1) were selected to be sequenced, adding to the three that were previously published. Taxa outside of *Paspalum* were selected to accommodate the available fossils within Poaceae [4]. Thus the analysis consists of the nine new plastomes, eight *Paspalum* species and *Paraneurachne muelleri*, and 45 other Poaceae plastomes from all subfamilies, which includes taxa for the divergence date analysis. The sampling also includes taxa in which mtDNA was found in the plastome, whether it is located in the *trnI*–*trnL* region [Parianinae: 9, 14] or in other regions [*Triticum*: 11].

**Table 1:** Insert size, IR size, and plastome size of *Paspalum* species and *Paraneurachne muelleri* with their vouchers and Genbank accession numbers. Newly sequenced plastomes are in bold

Taxa	Insert Size (bp)	IR Size (bp)	Plastome Size (bp)	Voucher^a^	Accession
***Paraneurachne muelleri***	2808	22,774	140,886	[19]	MG524000
*Paspalum dilatatum*	976	21,022	135,950	[3]	NC_030614
*P. fimbriatum*	2914	23,711	140,804	[3]	NC_030495
*P. glaziovii*	0	22,769	139,255	[3]	NC_030496
***P. inaequivalve***	0	22,783	140,312	PI 508769	MG524001
***P. ionanthum***	11,393	34,039	162,086	PI 404449	MG524002
***P. juergensii***	1012	21,968	137,513	PI 508779	MG523994
***P. minus***	6500	29,160	152,184	PI 404465	MG523995
***P. pubiflorum***	10,619	31,803	157,521	PI 304147	MG523996
***P. simplex***	3678	24,848	143,254	[19]	MG523997
***P. vaginatum***	0	22,777	140,451	[19]	MG523998
***P. virgatum***	3204	24,396	142,796	PI 364978	MG523999

^a^PI = Plant Introduction number, U.S. National Plant Germplasm System (https://www.ars-grin.gov/npgs/)

### Extraction and library preparation

DNA extractions from species of *Paspalum* were performed on young green leaf material dried in silica or herbarium specimens using the DNeasy Plant Mini kit (Qiagen, Valencia, CA, USA) according to the manufacturer’s instructions after a liquid nitrogen homogenization step. Samples were then prepared for NGS using the Illumina Nextera protocol. DNA samples were diluted to 2.5 ng/ul (50 ng total) and paired-end libraries were prepared using the Nextera DNA Sample Preparation kit. All libraries were sequenced at the core DNA facility at Iowa State University (Ames, Iowa, USA) on an Illumina HiSEq 3000. The sequence reads, from Washburn et al. [19], for *Paspalum simplex* (SRR2162764), *Paspalum vaginatum* (SRR2163016), and *Paraneurachne muelleri* (SRR2163452) were retrieved from the SRA archive at NCBI (https://www.ncbi.nlm.nih.gov/sra) for whole plastome assembly.

### NGS Plastome assembly, verification and annotation

Illumina and SRA reads were filtered and assembled following the methods of Burke et al. [3]. Reads of low quality were removed at default settings (DynamicTrim, SolexaQA++; [30]), excising adapters that were still attached to the reads (CutAdapt; [31]) and then the discarding of any read less than 25 base pairs (bp) long [LengthSort, SolexaQA++; 30]. SPAdes v3.6.1 [32] was used for de novo assembly with *k*-mers set from 19 to 73 bp with intervals of six. CD-Hit v4.6 [33] removed redundant sequences from the contig file. ACRE [34] was used to scaffold contigs together.

ACRE scaffolds and clean reads were imported into Geneious Pro v9.1.6 [35]. For each new accession, a closely related reference plastome, banked at NCBI, was chosen. The scaffolded contigs were then aligned to the reference plastome using the MAFFT v7.222 [36] plugin in Geneious. The gaps between the contigs were closed by using the map to reference function in Geneious to in silico genome walk [3]. A final verification of each plastome was performed by mapping the reads to the finalized plastome and manual adjustments were made when incongruences between the reads and the plastome occurred.

Verified plastome accessions were then pairwise aligned to their reference plastome, and annotations were applied using the transfer annotation feature in Geneious Pro. Coding sequence boundaries were inspected and manually adjusted to preserve reading frames. The inverted repeats (IRs) were located using the methods of Burke et al. [37]. BLASTn [38] was used to locate IR boundaries by aligning the new plastome to itself, and looking for segments in which the orientation of the reads transition from plus/plus to plus/minus. These boundaries were then flagged using the motif feature in Geneious Pro, and annotations for the IR were made.

### Phylogenomic analyses and divergence date estimation

A 61 taxa matrix of Poaceae plastid DNA (ptDNA) was assembled containing nine new plastomes and 11 *Paspalum* species overall. An alignment of these 61 complete plastomes, excluding one IR copy, was created using the MAFFT v7.222 [36] plugin in Geneious Pro, with default settings.

The *trnI*–*trnL* regions from species of *Paspalum* were extracted from the MAFFT alignment and manually aligned. This alignment was manually examined for shared or unique rare genomic changes and sequences with unexpected homologies were identified by BLASTn searches. Manual searches were also performed in this region to find sequence evidence for molecular mechanisms indicative of insertions, deletions, or site-specific recombinations. Such searches were also conducted on all pairwise alignments (55, total) of the *trnI* - *trnL* insert region of *Paspalum* spp. In particular, the presence of tandem repeats, dispersed repeats, and inverted repeats was investigated. Intermediate results suggested the presence of significantly placed dispersed repeats in the *trnI – trnL* insert region. Dispersed repeats were localized to endpoints of subregions of the *trnI* - *trnL* insert region via the motif search option in the Annotate and Predict menu in the sequence viewer of Geneious Pro. Searches were conducted from a minimum sequence length of five bp for dispersed repeats that mediate intrastrand deletions as suggested by Graur et al. [39].

Prior to plastome phylogenomic analyses of the 61 taxon matrix, any gaps that were produced by the alignment were then stripped from the matrix. A model for the stripped nucleotide alignment was selected using jModelTest v2.1.10 [40], and the GTR + I + G model was selected under the Akaike information criterion [41]. Maximum Likelihood (ML) and Bayesian MC3 Inference (BI) analyses were conducted on the alignment. The ML analysis was done via RAxML-HPC2 on XSEDE v8.2.9 [42] on the CIPRES Science Gateway [43]. The number of bootstrap replicates was set to 1000, and all other parameters were set to default. The BI analysis used MrBayes on XSEDE v3.2.3 [44] at the CIPRES Science Gateway. Settings for two independent analyses with four chains of twenty million generations each were specified, with a default burn-in value of 25%. The model was set to “invgamma” and “nst = 6”, with all other parameters at default.

An estimation of divergence times was performed on the 61 taxa stripped alignment with one IR in BEAST v2.4.3 [45]. Parameters included an uncorrelated relaxed clock model [46], a GTR substitution model with a gamma category count of six, an estimated shape parameter of 0.92 and an initial value of 0.52 for estimated proportion of invariant sites. Initial values for the shape parameter and proportion of invariant sites were obtained from the ML analysis.

The seven fossils that were chosen as calibration points are based on the recent use in divergence date analyses [4] and are reliably dated and confidently associated extant homologous species [47–49]. The specifics of these fossils are given (Additional file 1: Table S1). The fossil calibrations were placed on the assigned nodes (Additional file 1: Table S1) with the minimum node age set to the stratigraphic or radiometric date of the fossil and the maximum age set to the oldest known fossil in Poaceae [110 Ma; 49], generally following the methods of Christin et al. [46].

**Fig. 1:**
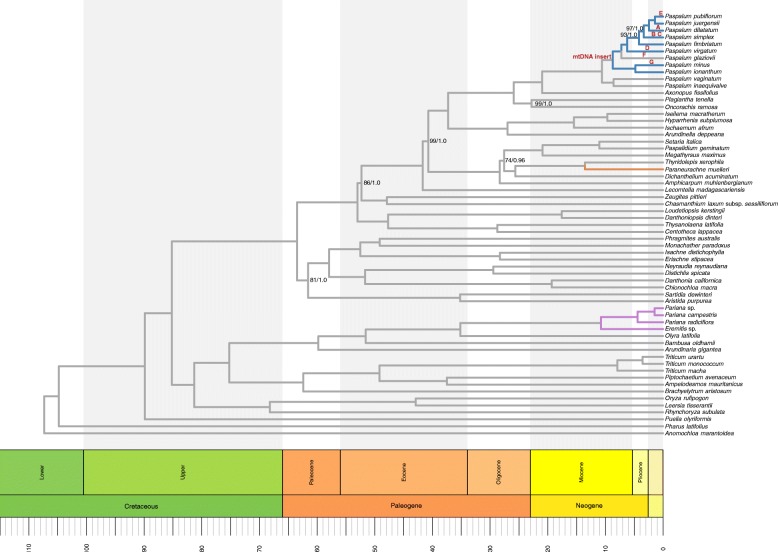
Chronogram of *Poaceae* species with support values (ML / BI) for nodes that did not recover maximum support. All other nodes are maximally supported. Highlighted branches depict different taxonomic groups with similar inserts in the *trnI* - *trnL* IGS. Letters in the *Paspalum* lineage designate IDEs (Table 2)

The BEAST analysis was conducted on the CIPRES Science Gateway for 40 million generations, logging at every 10,000 trees. Tracer v1.6 [50] was used to assess convergence. TreeAnnotator [51] was used to summarize the trees with a burn-in value of 25%. The plastome chronogram, with support values, was then visualized with the packages “strap” [52] and “ips” [53] in R [54]; (Fig. 1).

### Tetramer identity analysis

The contigs assembled in SPAdes for *Paspalum* were filtered in BLASTn at default parameters with a database containing the 11 published complete mitochondrial sequences from Poaceae (NC_007579.1, NC_008362.1, NC_011033.1, NC_007886.1, NC_007982.1, NC_029816.1, NC_013816.1, NC_008360.1, NC_008333.1, NC_008332.1, and NC_008331.1). The contigs, which showed high mitochondrial identity, were extracted using the BBmap “filterbyname.sh” executable script [55]. Five partitions were binned using Binning-Master [56] to determine tetramer identity with a sliding window of four nucleotides, advancing by one, for the Large Single Copy (LSC), Small Single Copy (SSC), IR without the insert, mitochondrial contigs retrieved from BLASTn filtering, and the *Paspalum* inserts in the IR. Files were then imported into Emergent Self-Organizing Maps (ESOM) software [57] to visualize the tetramers for each partition. Training was performed with the K-Batch algorithm of 20 epochs, with a starting radius of 50 and the dimensions of a 150 × 150 plot. The other parameters were set to default.

In contrast to pairwise comparisons, the ESOM method provides a more thorough and precise estimate of insert origin. The ESOM method statistically groups tetrameric sequence regions and determines if they have similar identities. Our hypothesis is as follows. If the *Paspalum* inserts were of plastid origin, then the tetramers would display strong elevation boundaries around other plastid regions: LSC, SSC and IR; otherwise they would be distributed among the mitochondrial sequences.

## Results

### Plastome features

GenBank accession numbers were obtained for the nine new complete plastomes in this study (Table 1). Not including the *trnI*-*trnL* IGS insert regions of the 11 *Paspalum* species, the overall pairwise identity was 98.3%, while the manually aligned *trnI*-*trnL* IGS insert region was 28.6% identical, due to the large number of gapped positions. The inserts in *Paspalum* ranged from 976 bp in *P. dilatatum* to 11,393 bp in *P. ionanthum*. Due to the length of this insert, *P. ionanthum* is now the longest known Poaceae plastome at 162086 bp. Other notable indels in the species of *Paspalum* were a 776 bp deletion in the *psbE-petL* IGS in all *Paspalum* except for *P. inaequivalve* and *P. vaginatum*. All Paspaleae shared a 222 bp deletion in the *petA-psbJ* IGS.

**Fig. 2:**
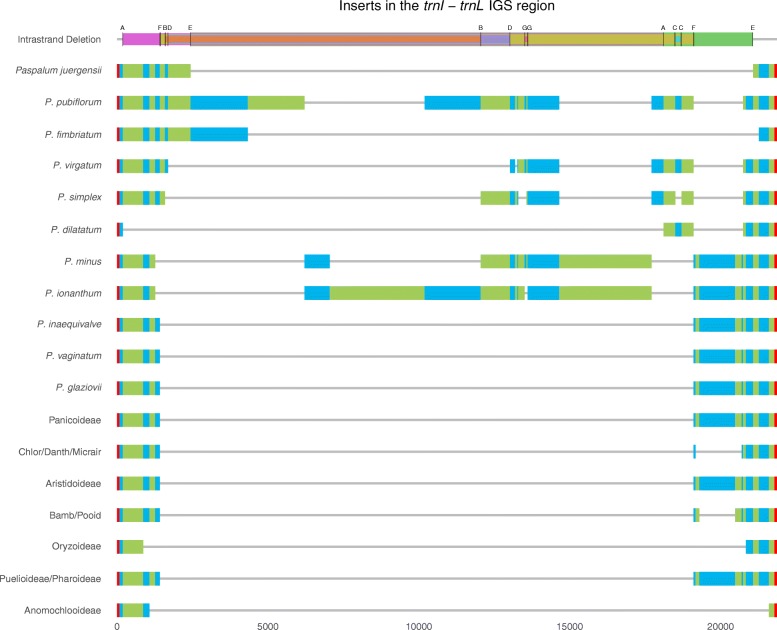
The inserts compared among *Paspalum* species and across generalized subfamilies. Aligned subregions are differentiated with alternating blue and green bars. These subregions are flanked by red bars representing *trnI* (left) and *trnL* (right). IDEs are represented with overlapping colored bars and their designated letter (Table 2)

Based on the manual alignment, 21 separate subregions in the *trnI*-*trnL* IGS insert were identified. Of these, 19 shared homology between two or more *Paspalum* species, and the other two, found in *P. publiforum* at 1878 bp and *P. ionanthum* at 3146 bp in length respectively, were unique to only those species. This was visualized (Fig. 2) using the function “geom_linerange” in ggplot2 [58] with the use of “select” and “gather” functions in tidyverse [59] to coerce the data, in the R statistical suite. Based on extensive searches of annotated regions, seven pairs of dispersed repeats were identified that immediately flanked presumed intrastrand deletion events (IDE). The size of the dispersed repeats ranged from 8 to 17 bp, and the deleted regions based on the alignment ranged from 112 bp, “IDE G,” to 18,645 bp, “IDE E” (Table 2). Note that since excisions via intrastrand deletion, which are mediated by recombination between repeats, eliminates one copy of the repeat in any given species, the existence of dispersed repeats was only identified when comparing homologous plastome regions between *Paspalum* sp. Finally, a BLASTn search of the concatenated *Paspalum* insert sequence representing the progenitor insert, 17,685 bp in length, returned the top hit of the complete mitochondrial genome of *Tripsacum dactyloides* (DQ984517) at 99% identity for 33% of the sequence.

**Table 2:** A list of intrastrand deletion events (IDEs) in the *Paspalum* lineage

IDE	Sequence	Deletion Length (bp)	Divergence Time (Ma)	*Paspalum* with Deletion	Both repeats present at each end of the deletion	One repeat present at one end of the deletion
A	CAAATATTCGGA	17,929	2.4	*P. dilatatum*	*P. pubiflorum, P. virgatum, P. simplex*	*P. juergensii, P. fimbriatum, P. minus, P. ionanthum, P. glaziovii, P. inaequivalve, P. vaginatum*
B	ATATAACTTCCC	10,466	3.3	*P. simplex*	*P. pubiflorum*	*P. juergensii, P. fimbriatum, P. virgatum, P. minus, P. ionanthum*
C	TCCTGAAACGTAAAAAA	220	3.3	*P. simplex*	*P. dilatatum, P. pubiflorum, P. virgatum*	
D	ACTTGCTC	11,340	6.2	*P. virgatum*	*P. pubiflorum*	*P. juergensii, P. fimbriatum, P. simplex, P. minus, P. ionanthum*
E	ATGATTTGG	18,645	1.4	*P. juergensii*	*P. pubiflorum*	*P. fimbriatum, P. dilatatum, P. virgatum, P. simplex, P. minus, P. ionanthum, P. glaziovii, P. inaequivalve, P. vaginatum*
F	GGAATCCGTTAGAAAT	17,700	2.0	*P. glaziovii, P. inaequivalve, P. vaginatum*		*P. dilatatum, P. juergensii, P. fimbriatum, P. pubiflorum, P. virgatum, P. simplex, P. minus, P. ionanthum,*
G	GGCTCGGCGAC	112	4.8	*P. ionanthum*	*P. pubiflorum, P. virgatum, P. minus*	*P. simplex*

The *trnI*-*trnL* IGS insert in *Paraneurachne muelleri* was 2808 bp long, and contained no similarity to the subregion found in *Paspalum* or Parianinae. BLASTn results returned a 92% shared identity to *Sorghum bicolor* mitochondrial genome (DQ984518.1) for 68% of the sequence.

### Plastome Phylogenomic and divergence date analyses

The same topologies were retrieved for the ML and BI analyses. Most nodes were supported maximally except for seven nodes in the ML analysis and one node in the Panicinae in the BI analysis (Fig. 1). In both analyses Panicoideae were sister to the rest of the PACMAD clade with a bootstrap value of 81% and a posterior probability (PP) of 1.0. The *Paspalum* species were retrieved as monophyletic, with only two bootstrap values being less than maximum and all had a PP of 1.0.

The same topology was retrieved in the divergence date analysis performed by BEAST v2.4.3. The crown node of the PACMAD clade was dated at 63.5 Ma with crown nodes of Panicoideae diverging at 53.0 Ma, Paspaleae at 25.9 Ma and Paspalinae at 21.0 Ma. The crown node of *Paspalum* diverged at 10.6 Ma, with the most recent diversification event at 1.4 Ma with the divergence of *P. pubiflorum* and *P. juergensii* from their common ancestor. The shortest time between species divergences is 0.9 Ma, from which *P. simplex* diverges from the sister clade comprised of: *P. dilatatum*, *P. pubiflorum*, and *P. juergensii*. The longest time between divergence events is 8.6 Ma with the bifurcation of *P. vaginatum* and *P. inaequivalve,* the earliest lineage in *Paspalum* sampled here.

### Tetramer identity analysis

The tetramer matrices were generated for each of the five partitions. These files were made into the U-Matrix, which is a visualization of distance in the tetramer frequency between data points and is represented as map elevation. These distances between clusters of points are further visualized with elevation barriers acting as cutoffs, thus representing large differences between data sets [56, 57]. This was used to interpret the origin of *Paspalum* inserts, depending on where they occurred and how they were separated in the map.

**Fig. 3:**
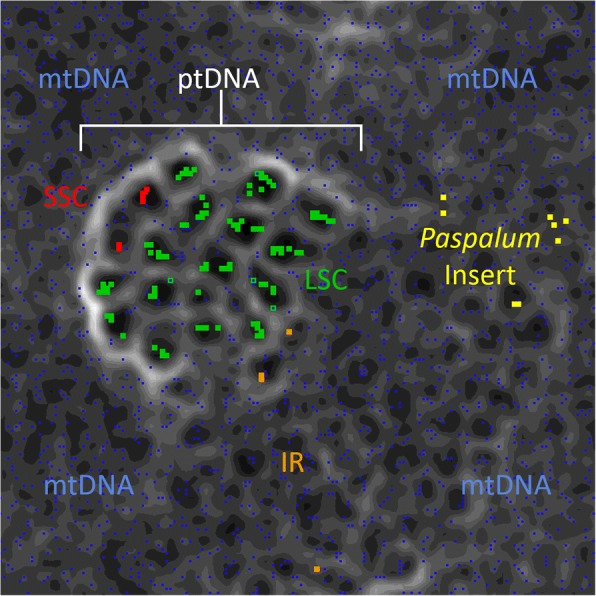
ESOM of mtDNA and partitions of ptDNA sequences based on tetranucleotide frequency. The mtDNA is in blue with the ptDNA divided into LSC (green), SSC (red), IR (orange), and the *Paspalum* inserts (yellow). High ‘elevations’ are in white visualizing major distances in tetranucleotide frequency, while ‘low elevations’ are in darker grays showing similarity in tetranucleotide frequency

Inspection of the ESOM results shows that most of the plastome clusters together. The LSC and SSC cluster with high ‘elevation’ (white and light grey areas) around most of the points, and with lower ‘elevation’ (dark grey and black areas) compartmentalizing clusters of LSC and SSC within that barrier. The IR is close to the LSC/SSC regions along an area where the elevation is not as high. There is one cluster of IRs that is not near LSC/SSC area, and is located among mtDNA with no high ‘elevation’ barriers nearby. The *Paspalum* inserts were also located within the mtDNA region, also in an area with very few high ‘elevation’ barriers. Some of the insert data points clustered, with two groups of two and one group of four. The mtDNA points were located throughout the ESOM, but with fewer data points located within the LSC/SSC area (Fig. 3).

## Discussion

### Plastome features

In the eight newly assembled *Paspalum* species, six contained an insert in the *trnI*-*trnL* IGS. We investigated whether the origin of the inserts was one or multiple events. Inserts from different species had little overall similarity and only one species, *P. fimbriatum*, had a secondary insert, which had homology to a smut parasite of grasses [3]. However, while none of the sequences are exactly the same for each species, these six new inserts contain multiple subregions of high identities that are shared between sections of newly and previously discovered *Paspalum* inserts [3]. Thus, 19 out of the 21 subregions (Fig. 2) contain shared sequence similarity across the sampled *Paspalum*, which suggests a single insertion event at some point in the *Paspalum* lineage (Fig. 1). With the current sampling, the hypothetical ancestral insert size is estimated to be around 17,685 bp long, and would have only occurred once. This is in agreement with previous characterizations of mitochondrial inserts in plastomes, which suggest that while these insert events happen, they are rare [25].

The homologous IGS also contained an insert for the newly sequenced *Paraneurachne muelleri*. While this 2808 bp insert did not match any subregion of the *Paspalum* inserts or the Parianinae inserts, it does illustrate that this IGS in the plastome is a hotspot for indel mutations. On average the lengths of plastid IGSs for all Poaceae in this sampling, removing species with probable mitochondrial inserts, is 422 bp, yet the length of the *trnI-trnL* IGS is on average 3071 bp. The greater length of this IGS is likely due to the disintegration of the large *ycf2* CDS, which is one of the defining features for poaceous plastomes [60]. The larger size of the plastome IGS potentially offers less steric hindrance during recombination with the larger mitochondrial insert. Future discoveries of more grass genera with insertions in this *trnI* - *trnL* IGS, would support the idea of this spacer as a mutational hotspot for recombination mutations.

The most likely mutational mechanism that can explain the varying lengths of these inserts is gene conversion followed by repeated rounds of intrastrand deletions. Gene conversion, or nonreciprocal recombination, occurs when homologous regions have uneven replacement of sequence, causing the loss of one variant sequence [39]. To help put this mechanism in context, an understanding of plant mitochondrial genomes is warranted.

Mitochondria are known to actively take up and incorporate foreign DNA, which includes ptDNA [61]. One study [62], found that 22,593 bp of ptDNA was located in a mitochondrial genome (AB076665–6). That only accounts for 6.3% of the mitochondrial DNA, but it is nearly 20% of the donor plastome (NC_001320). Another study [63], found two major ptDNA inserts, of 12.6 kb and 4.1 kb that were primarily IR sequence, and with other inserts totaled 23.9 kb. This only accounts for 4.2% of the mitochondrial genome (AY506529), but is 17.0% of the respective plastome (X86563). Within these plastome inserts in mitochondrial genomes, complete genes can be found like *trnI* (NC_007982 at 326704–327726) and *trnL* (NC_007982 at 336020–336100). Since mitochondrial genomes are known for rearranging to the extent that gene order is not conserved between genera or even congeneric species [64–67], it is likely that the intergenic distance of genes in the mitochondrial genome with homology to those in the plastome would differ in length and sequence between grass taxa.

Thus, sequences in the mitochondrial genome that contain mtDNA flanked by conserved regions of ptDNA (*trnI* and *trnL*) would have the potential for gene conversions. The conserved ptDNA would act as recombination points between the plastome loci and homologous loci in the mitochondrial genome to create mtDNA insertions. The varying lengths seen among different genera are also explained by rearrangements within the mitochondrial genome that are specific to a taxonomic level, such as within the *Paspalum* or Parianinae lineages. This creates varying insert lengths and with sequence content unique to the separate insertion events at different taxonomic levels (*Paspalum* progenitor: 17685 bp, *Paraneurachne*: 2808 bp, Parianinae: 4920 bp).

Once the inserts are established in the plastome, the mechanism for the differential degradation that is seen within *Paspalum* can easily be explained. The identification of dispersed repeats localized to the endpoints of these areas clearly indicate IDE as the causal mechanism. There are seven examples of IDEs (Table 2) that have removed sequences of varying lengths, ranging from 112 bp to 18,645 bp, throughout the insert subregions in *Paspalum* and flanking ptDNA, which precisely terminate in short dispersed repeats. These IDEs, with other probable ones that are undetectable due to the taxonomic subset that was sampled, create sequences that are seemingly unique, but are just the result of various ways to excise sequence from the original insert.

An IDE may also explain a singular situation for one species, *P. glaziovii,* which does not contain mtDNA inserts. There is evidence for an IDE within *Paspalum* that can remove the entire insert. An upstream repeat (16 bp in length) can be found early in the sequence, near *trnI*, for *P. juergensii*, *P. fimbriatum*, *P. pubiflorum*, *P. virgatum*, and *P. simplex*, while a downstream exact repeat is found closer to the *trnL* gene in *P. minus, P. ionanthum, P. glaziovii, P. inaequivalve,* and *P. vaginatum*. At this point there is no *Paspalum* species that has both copies of this repeat. In those species that contain one repeat copy and insert sequence, it is likely that a smaller IDE removed one of the repeats. Doing so would preserve a larger portion of the original insert, and potentially create contingencies on what other IDEs can occur based on whether the first or second repeat was removed.

The hypothesized smaller IDEs that preserved the insert by removing one of the repeats appears to have also removed the possibilities of other IDEs. An example of this is at the beginning of *P. minus,* and *P. ionanthum,* which are missing one subregion prior to “IDE F,” likely due to a smaller IDE. This unidentified IDE most likely removed not only the first repeat of “IDE F,” but also the first of the dispersed repeats for “B”, “D”, and “E”. Thus, the contingency of future IDEs based on a previous IDE and understanding priority of IDEs will be better determined with further sampling within the *Pasplaum* lineage (for example Piot et al. [10], and future studies).

### Phylogenomic and divergence date analyses

The topology of *Paspalum* species for this study was well supported with all but two nodes being maximally supported in the ML analysis, and all nodes maximally supported in the BI analysis. With little doubt as to the divergence of the represented species, we find that species with no evidence of an insert are non-monophyletic. *P. inaequivalve* and *P. vaginatum*, which both lack the insert, are in the earliest diverging clade in this study, but *P. glaziovii*, which also lacks the insert, is more recently diverged within the *Paspalum* lineage. This demonstrates that the lack of an insert is not an ancestral trait. Based on the current sampling, it is likely the insert arose at the clade comprising all *Paspalum* except *P. inaequivalve* and *P. vaginatum*. This might explain why *P. ionanthum* has the longest insert (11,393 bp), as it is near the origin of the insertion event. With the likely point of origin for the insertion, determining the time from foreign DNA insertions to IDEs will further the understanding of how plastomes interact with inserted non-ptDNA.

In the chronogram phylogeny, selected divergence dates for speciation effectively represent the hypothetical time of the insertion event and the time it takes for IDEs to become fixed in the plastome. If the insert arose around 8.7 Ma, and the subsequent loss of the insert in *P. glaziovii* occurred at 7.2 Ma, then the fixation of “IDE F” occurred within a time period of roughly 1.5 Ma. Other IDEs (A, B, D, E, F), became fixed in relatively short time periods as well, ranging from as little as 1.4 Ma (“IDE E”) to 3.3 Ma, except for “IDE D” at 6.2 Ma. These larger time estimates could be due to a lack of sampling, and not being able to break up longer branches with a species rich genus like *Paspalum* (~ 350 spp.) [29]. Thus, IDEs, especially larger ones, becoming fixed in a relatively short time could suggest that the original insert is being selected against.

The progenitor’s hypothesized insert size is over 17,000 bp, and contains no recognizable coding sequence. The longer the insert, the more time, energy and materials will be needed in the replication process of the plastome. This might explain for the variety of different IDEs seen throughout the phylogeny. While some species of *Paspalum* are missing one or both of the repeats that can remove the insert, “IDE F,” they contain other IDEs that either shorten the insert or remove most of the insert with the ptDNA that flanks the insert (*P. juergensii*, *P. fimbriatum*, and *P. dilatatum*). Thus, a smaller plastome, with little or no insert, could be more beneficial overall to the plastid compared to larger plastomes.

### Tetramer identity analysis

It is also important to determine the origin of the insert as it could have other implications if the source is not from mtDNA. For this, the ESOM analysis (see visual matrix output, Fig. 3) was conducted to compare tetranucleotide frequencies to evaluate support for our hypotheses of origin. The *Paspalum* insert within the ESOM matrix did not cluster with other ptDNA, but rather clustered into smaller groups among the mtDNA. From the ESOM matrix (Fig. 3), there is a distinct high ‘elevation’ around the ptDNA. This circumscription of ptDNA is more apparent near the top and left side portion of the ptDNA cluster, with the loss of ‘elevation’ as it gets closer to the sections with IR sequences. This can be explained as there are large portions of IR that are located in the mtDNA of many Poaceae species [62, 63]. Thus, there is some similarity creating a lower ‘elevation’ around IR points. The incorporation of ptDNA into mtDNA is also why there are points from the mtDNA within the ptDNA cluster [62, 63]. There is also one IR point that is noticeably distant from the rest of the ptDNA cluster. This is most likely the rRNAs that are found within the inverted repeats. These regions are very GC rich (*Paspalum* and other Poaceae species in this study: GC% = 54.7%) and thus would look more like mtDNA, which is overall also more GC rich (AY506529: GC% = 43.9%, AB076665–6: GC% = 43.9%) than the overall AT rich ptDNA (*Paspalum*: GC% = 37.8%, Poaceae species in this study: GC% = 37.6%).

Our interpretation of these results is that the insert is mtDNA in origin, which was then transferred into ptDNA by mechanisms suggested previously. This possibility, which was once thought to be impossible or highly unlikely [23, 24], is only now becoming evident as more NGS work is done [3, 9, 10, 14, 28].

## Conclusion

In conclusion, the *Paspalum* insert in the *trnI* - *trnL* IGS was observed in eight out of the eleven banked plastomes. The original insert was determined to have similarities to mtDNA suggesting mtDNA origins. This is supported by the high pairwise identity compared to other mitochondrial plastomes, and the placement of the insert in the tetranucleotide analysis. It is likely that this mtDNA insert arose at the clade comprising all *Paspalum* except possibly *P. inaequivalve* and *P. vaginatum* around 8.7 Ma, and mtDNA sequence was subsequently lost in certain species. The deletion events in the insert were not always complete removals, but there is a general trend of removing large subregions of sequence. This is seen throughout the *Paspalum* lineage, with seven different IDEs identified. These IDEs remove large portions of sequence in a given species lineage within a relatively small amount of time, the shortest of which was noted at 1.4 Ma. Thus, this suggests that there might be evolutionary pressure to remove this excess mtDNA from the plastome.

## Additional file

Additional file 1: **Table S1** Information of fossils calibrations used for divergence date analysis [49, 68–73]. (DOCX 14 kb)

## Abbreviations

ACRE: Anchored conserved region extension
BEAST: Bayesian Evolutionary Analysis Sampling Trees
BI: Bayesian MC3 Inference
BLASTn: Nucleotide Basic Local Alignment Search Tool
bp: Base pair
CIPRES: Cyber infrastructure for phylogenetic research
contigs: Contiguous sequences
ESOM: Emergent Self-Organizing Maps
GTR + G + I: General time reversible plus gamma distribution plus proportion of invariant sites
IDE: Intrastrand deletion event
IGS: Intergenic spacer
IR: Inverted repeat
Kbp: Kilobase pairs
LSC: Large single copy
Ma: Megaannus or million years
MAFFT: Multiple alignment using fast fourier transform
ML: Maximum likelihood
mtDNA: Mitochondrial DNA
NCBI: National Center for Biotechnology Information
NGS: Next generation sequencing
PACMAD: Panicoideae Arundinoideae Chloridoideae Micrairoideae Arundinoideae Danthonioideae
PP: Posterior probability
ptDNA: Plastid DNA
SSC: Small single copy
