# Supplementary material for: Investigation of mitochondrial-derived plastome sequences in the Paspalum lineage (Panicoideae; Poaceae)
Source: BMC Plant Biol. 2018 Aug 3;18:152. doi: 10.1186/s12870-018-1379-1 (PMC6091044; doi:10.1186/s12870-018-1379-1)
Supplement: Supplementary file 1 — Table S1 Information of fossils calibrations used for divergence date analysis [49, 68–73]. (DOCX 14 kb) [file 12870_2018_1379_MOESM1_ESM.docx]

Additional file 1 Table S1 Information of fossils calibrations used for divergence date analysis.

| Fossil | Fossil type/Evidence | Age/Lower Bound (Ma) | Assigned node^a^ | Citations |
| --- | --- | --- | --- | --- |
| Spikelet clade, unspecified | Spikelet in amber | 97 | Spikelet clade | [49] |
| Oryzeae | Phytoliths | 66 | Oryzoideae | [68] |
| Stipa florissanti | Fruits | 34 | (*Piptochaetium*, *Ampelodesmos*) | [69] |
| Leersia seifhennersdorfensis | Inflorescence | 30 | (*Leersia*, *Oryza*) | [70] |
| Distichlis sp. | Leaf fragments | 14 | (*Distichlis* *spicata*, *Neyraudia*) | [71] |
| Dichanthelium | Fertile lemmas and paleas | 8 | (*Dichanthelium*, (*Paraneurachne*, *Thyridolepis*)) | [72] |
| Setaria | “Seeds” | 7 | (*Setaria*, *Paspalidium*) | [73] |

^a^All assigned nodes are crown nodes.
